# Effects of epidermal growth factor receptor kinase inhibition on radiation response in canine osteosarcoma cells

**DOI:** 10.1186/s12917-016-0707-7

**Published:** 2016-05-31

**Authors:** Fernanda B. Mantovani, Jodi A. Morrison, Anthony J. Mutsaers

**Affiliations:** Department of Clinical Studies, Ontario Veterinary College, University of Guelph, Guelph, Ontario Canada; Department of Biomedical Sciences, Ontario Veterinary College, University of Guelph, Guelph, Ontario Canada

**Keywords:** Osteosarcoma, Dog, Canine, Erlotinib, Epidermal growth factor receptor (EGFR), Radiation, Vascular endothelial growth factor (VEGF), Radiosensitization

## Abstract

**Background:**

Radiation therapy is a palliative treatment modality for canine osteosarcoma, with transient improvement in analgesia observed in many cases. However there is room for improvement in outcome for these patients. It is possible that the addition of sensitizing agents may increase tumor response to radiation therapy and prolong quality of life. Epidermal growth factor receptor (EGFR) expression has been documented in canine osteosarcoma and higher EGFR levels have been correlated to a worse prognosis. However, effects of EGFR inhibition on radiation responsiveness in canine osteosarcoma have not been previously characterized. This study examined the effects of the small molecule EGFR inhibitor erlotinib on canine osteosarcoma radiation responses, target and downstream protein expression in vitro. Additionally, to assess the potential impact of treatment on tumor angiogenesis, vascular endothelial growth factor (VEGF) levels in conditioned media were measured.

**Results:**

Erlotinib as a single agent reduced clonogenic survival in two canine osteosarcoma cell lines and enhanced the impact of radiation in one out of three cell lines investigated. In cell viability assays, erlotinib enhanced radiation effects and demonstrated single agent effects. Erlotinib did not alter total levels of EGFR, nor inhibit downstream protein kinase B (PKB/Akt) activation. On the contrary, erlotinib treatment increased phosphorylated Akt in these osteosarcoma cell lines. VEGF levels in conditioned media increased after erlotinib treatment as a single agent and in combination with radiation in two out of three cell lines investigated. However, VEGF levels decreased with erlotinib treatment in the third cell line.

**Conclusions:**

Erlotinib treatment promoted modest enhancement of radiation effects in canine osteosarcoma cells, and possessed activity as a single agent in some cell lines, indicating a potential role for EGFR inhibition in the treatment of a subset of osteosarcoma patients. The relative radioresistance of osteosarcoma cells does not appear to be related to EGFR signalling exclusively. Angiogenic responses to radiation and kinase inhibitors are similarly likely to be multifactorial and require further investigation.

## Background

Osteosarcoma (OSA) is the most common primary bone tumor of the domestic dog, occurring predominantly in large breeds, and accounting for up to 85 % of skeletal tumors in this species [1]. Local tumor growth causes severe pain and lameness secondary to bone lyses, proliferation or both, and eventual metastasis from OSA to the lungs and other locations occurs in the vast majority of cases [1]. Surgical removal of the primary tumor, either by amputation of the affected limb or by limb-sparing surgery, followed by adjuvant chemotherapy is considered the standard of care for canine OSA. However, surgery may be contraindicated in dogs with preexisting orthopedic or neurologic disease, may not be elected by owners, or may not be feasible in cases of tumors affecting the axial skeleton. Thus, there is increasing interest in treating the primary tumor by utilizing external beam radiation therapy (RT) for dogs with OSA. Radiation therapy has mainly been applied in palliative settings to provide analgesia and improve quality of life for canine OSA patients. Most reports in the veterinary literature describe radiation protocols consisting of two to four treatments (fractions), delivering total doses of 16 to 32 Gray (Gy) [2]. Although pain control is achieved in approximately 70–90 % of treated dogs, responses seen with palliative RT protocols are transient, with clinical improvement lasting approximately 2 to 4 months [2]. Treatment failure is associated with recurrent primary tumor growth and therefore novel strategies to improve the response to RT for canine OSA may translate into better clinical outcomes for these patients. Pre-clinical work conducted in vitro using cell lines has indicated that canine OSA is a moderately radioresistant tumor, with a high mean surviving fraction after treatment with 2 Gy [3]. Increasing the sensitivity of OSA cells to ionizing radiation could enhance the effects of RT, possibly improving patient outcomes.

Advances in molecular biology have resulted in the identification of several pathways involved in the pathogenesis and progression of cancer, which can be utilized as therapeutic targets. The epidermal growth factor receptor (EGFR) is a transmembrane receptor tyrosine kinase (RTK) involved in signaling for cell growth, proliferation, invasion and survival [4]. Over-expression and constitutive activation of EGFR have been found in numerous human cancers, including breast, lung and head and neck carcinomas [5]. In veterinary oncology, EGFR expression has been identified in various epithelial malignancies, including canine lung, nasal, mammary and transitional cell carcinoma, and feline squamous cell carcinoma (SCC) [6–11]. Additionally, higher expression levels of EGFR have been associated with more aggressive cancer behavior [6–11]. The role of aberrant activation of EGFR in the pathogenesis of mesenchymal tumors, such as OSA, is less well defined. Expression of EGFR has been documented in human [12, 13] and canine OSA [14], and correlated with a worse prognosis, indicating that EGFR may play a role in OSA tumor biology and therefore EGFR pathway inhibition could represent a viable treatment option for OSA. In vitro targeting of EGFR with RTK inhibitors has been reported in the veterinary literature, with successful inhibition of cell proliferation and growth of canine mammary carcinoma and OSA cell lines [15, 16], further supporting EGFR inhibition as a possible treatment approach for canine OSA.

The combining of RT with cytotoxic chemotherapy and/or more targeted cancer therapeutics has been widely investigated in human oncology, with the goal of improving the effectiveness of radiation (radiosensitization) [4]. Targeting the EGFR pathway is an attractive approach for radiosensitization for multiple reasons. EGFR inhibitors commonly produce a cytostatic effect with arrest in the G1 phase of the cell cycle, which can prevent tumor cell repopulation post-radiation [17, 18]. Additionally, exposure of tumor cells to ionizing radiation can activate EGFR independently from ligands, contributing to tumor radioresistance [4, 19, 20]. Therefore, neutralizing this tumor response to radiation by inhibiting EGFR signaling could maintain tumor sensitivity. Erlotinib is a selective inhibitor of EGFR tyrosine kinase, which blocks cell cycle progression at the G1 phase and induces apoptosis of select human carcinoma cells in vitro [21]. Erlotinib has been used in the treatment of several human malignancies, and is approved for the treatment of non-small-cell lung cancer (NSCLC) and advanced pancreatic cancer in the United States. In human oncology, the use of erlotinib as a radiosensitizer has been successful in pre-clinical work [22–24], and has shown promising results in phase I/II clinical trials for head and neck SCC and NSCLC [25–27].

The effects of EGFR activation are exerted via subsequent activation of multiple downstream intracellular signaling pathways, including the phosphatidylinositol-3-kinase (PI3K) signaling cascade that culminates with activation of the serine/threonine kinase Protein kinase B (PKB/Akt). Upon stimulation of EGFR, PI3K is activated and generates phosphatidylinositol-3,4,5-trisphosphate (PIP3), which in turn acts as a second messenger for activation of Akt. Upon activation, Akt phosphorylates numerous downstream cytoplasmic and nuclear substrates, ultimately resulting in enhanced cell survival, proliferation, and inhibition of apoptosis [28, 29]. Radiation treatment may lead to enhancement of this signaling pathway in cancer cells as a response to treatment. Exposure of human carcinoma and glioblastoma cells to radiation in vitro activated Akt, and promoted increased cell survival and proliferation [28–30], through activation of EGFR via a ligand-independent mechanism. In these studies, increased levels of phosphorylated-Akt (p-Akt) were found within 4 h of RT, and inhibition of Akt enhanced radiosensitivity of tumor cells [28–30]. It is possible that similar EGFR activation and secondary increases in p-Akt levels could be seen following RT of canine OSA. Furthermore, evaluating the PI3K/Akt pathway could potentially serve as a surrogate biomarker for inhibition of upstream receptor targets like EGFR after treatment with erlotinib or other agents.

This study investigated the effects of erlotinib alone and in combination with RT on canine OSA cell lines. Therapeutic effects were evaluated by clonogenic survival, cell viability, and the expression of target and downstream proteins. Finally, because one of the mechanisms of action for both radiation and EGFR inhibition has been shown to be inhibition of angiogenesis, we investigated the impact of treatment on levels of the potent angiogenesis factor vascular endothelial growth factor (VEGF) secreted by OSA cells into conditioned media. Dose dependent erlotinib single agent activity was observed in all cell lines. Erlotinib provided enhancement of radiation effects on Dharma OSA cells at 2, 4 and 6 Gy doses, which are lower doses than the commonly used 8 Gy per fraction dose utilized in most palliative radiation protocols for osteosarcoma. Erlotinib increased VEGF levels in conditioned media and this effect was particularly evident with combination treatment.

## Methods

### Cell culture

Canine osteosarcoma cell lines D17, Abrams and Dharma were used. D17 cells were obtained from Sigma-Aldrich/European Collection of Cell Cultures (ECACC). Abrams cells were a generous gift from Mike Huelsmeyer at the University of Wisconsin. Both D17 and Abrams cell lines have been utilized on several published studies and have been characterized as canine OSA cells based on morphology and xenograft analysis [31]. Dharma cells were isolated and adapted to culture from a clinical case by Dr. Anthony Mutsaers, and validated as OSA by histopathology evaluation of tumors produced from successful xenograft outgrowth after implantation in immunocomprised (nude) mice. All cells were grown in Dulbecco’s modified Eagle’s media (Hyclone DMEM - Fisher Scientific- Ottawa, ON, Canada) supplemented with 10 % fetal bovine serum (Life Technologies, Burlington, ON, Canada) and 1 % penicillin/streptomycin (BioWhittaker, Mississauga, ON, Canada). All cell cultures were maintained at 37 °C and 5 % CO2 in a humidified incubator.

### Radiation therapy

Cell culture plates were irradiated at ambient temperature and pressure, at a rate of 400 monitor units/min utilizing a 6-MV linear accelerator (Clinac IX System, Varian Medical Systems, Inc., Palo Alto, CA, USA). Cell culture dishes were placed between two solid water-equivalent plates, with thickness of 4.5 cm on top and 5 cm on the bottom. The dose distribution for this set up was medical physicist verified. Control cell culture plates were transported to the radiation therapy area but kept outside the radiation vault during treatments.

### Clonogenic survival

Cells were seeded into six-well plates (D17 and Abrams at 500 cells/well, and Dharma at 1,500 cells/well) with 3 ml of media. After 24 h, the media of all wells was replaced and erlotinib (SelleckChem, Houston, TX, USA) at 10 μM was added to treatment group wells. Erlotinib was diluted in dimethyl sulfoxide (DMSO) resulting in a final concentration of 0.04 % DMSO in each well. After incubation for 4 to 6 h, doses of 0, 2, 4, 6, 8 and 10 Gy of radiation were administered to individual plates. Colony formation was monitored daily and the experiment stopped after 10 to 14 days, before the control colonies became confluent. Cells were stained with 0.5 % crystal violet in 20 % methanol for 30 minutes, then washed gently twice with tap water [32]. Colonies were visualized by light microscopy and counted. A colony was defined as an aggregate of ≥ 50 cells. The cell surviving fraction, normalized for plating efficiency, was determined for each radiation dose. All experiments were repeated three times.

### Cell viability

To assess cell viability, Resazurin Cell Viability Kit (Sigma-Aldrich, Oakville, ON, Canada) was used at a concentration of 5.0 mg/ml. Cells were seeded into 96-well plates (D17 and Abrams at 500 cells/well, and Dharma at 2,000 cells/well), and settled for 24 h at 37 °C and 5 % CO2 [33]. Erlotinib was administered at 10 μM and 40 μM, plates were incubated for 4 to 6 h, and doses of 0, 2, 4, 6, 8 and 10 Gy of radiation were delivered to individual plates. After 72 h, 100 μl of Resazurin solution was pipetted into each well. After the solution in wells changed in color, absorbance readings were obtained from a Synergy 2 spectrophotometer (BioTek, Winooski, VT, USA), at an excitation wavelength of 570 nm and emission wavelength of 600 nm. Relative viable cell number was assessed by means of sextuplicate wells for each erlotinib concentration and corresponding control group, and each experiment was repeated three times. Absorbance values were corrected for media only readings in sextuplicate wells.

### Protein detection

Cells were seeded into six-well plates (D17 and Abrams at 150,000 cells/well, and Dharma at 200,000 cells/well) and settled for 24 h at 37 °C and 5 % CO2. The media of all wells was replaced to divide groups into erlotinib at 10 μM or control, followed by incubation for 4 to 6 h. Plates were irradiated with a 2 Gy dose or kept in the radiation control room during treatment. Cells were lysed in ice cold buffer (Cell Signaling technology, Whitby, ON, Canada) containing aprotinin, phenylmethanesulfonyl fluoride and a phosphatase inhibitor cocktail, and collected 0.25, 0.5, 1, 2, 24 and 48 h post radiation, and placed immediately on ice. Cell lysis buffer additives were obtained from Sigma-Aldrich (Oakville, ON, Canada). Equal amounts of protein were separated by SDS polyacrylamide gel electrophoresis and transferred to a polyvinyl difluoride membrane (Roche Diagnostics Corporation, Indianapolis, IN, USA). Membranes were hybridized to an appropriate primary antibody and horseradish peroxidase (HRP) conjugated secondary antibody, then visualized using the Bio-Rad Chemi-Doc system (Universal Hood III). Primary antibody against β-actin, EGFR, Akt and p-Akt were purchased from Cell Signaling Technology (Whitby, ON, Canada). The secondary antibodies, HRP-conjugated goat anti-rabbit IgG were obtained from Santa Cruz Biotechnology Inc. (Dallas, TX, USA).

### VEGF levels

Conditioned media was collected and pooled from sextuplicate wells treated with erlotinib at 10 μM, with or without radiation treatment at 2 Gy and 8 Gy at 72 h. Levels of VEGF were quantified using the Quantikine Canine VEGF ELISA Kit (R&D Systems, Minneapolis, MN, USA), following the manufacturer’s instructions [33]. The optical density of the standard solutions was plotted against their corresponding concentrations to generate a standard curve and allow determination of sample VEGF concentrations. Absorbance was read at 450 nm and corrected by subtracting readings at 540 nm, as per manufacturer recommendation.

### Statistical analysis

Statistical analyses were performed with Graph-Pad Prism 5 software (GraphPad Software, Inc., La Jolla, CA, USA). For clonogenic survival and cell viability assays, a two-way analysis of variance (ANOVA) with Sidak method for multiple comparisons was used to determine whether erlotinib treatment had an effect in clonogenic survival and cell viability compared to radiation only treatment groups. For VEGF levels, a one-way ANOVA was used to determine whether treatment with erlotinib and/or radiation had an effect on VEGF concentrations compared to control groups. To account for changes in cell number that may influence VEGF levels, readings were normalized to cell viability of respective wells, as measured by Resazurin assay. Overall significance was set at *p* < 0.05.

## Results

### Effects of erlotinib and radiation on clonogenic survival

**Fig. 1:**
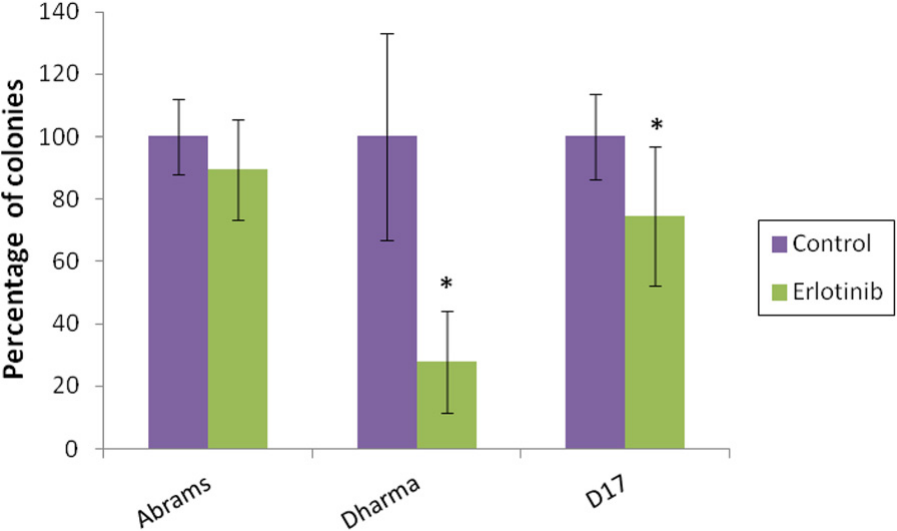
Effects of single agent erlotinib on clonogenic survival. Canine OSA cells treated with erlotinib at 10 μM for 4-6 h (“erlotinib”). Experiments were repeated three times and average of results are shown. Erlotinib showed single agent activity through reduction in clonogenic survival in 2 out of 3 cell lines. * *p* < 0.05 indicates statistical significant reduction in clonogenic survival compared to control

**Fig. 2:**
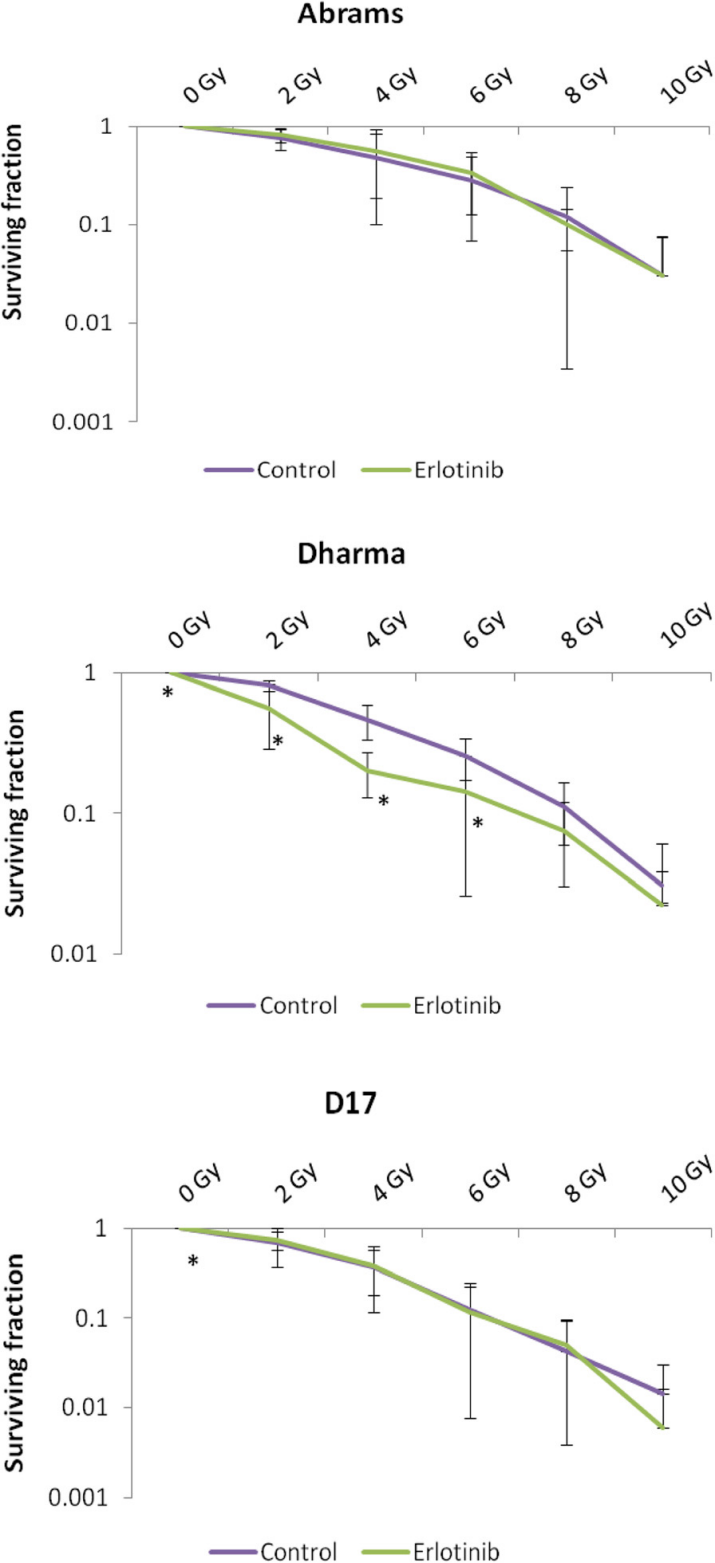
Clonogenic survival curves. Canine OSA cells were treated with radiation only (“control”) or in combination with erlotinib at 10 μM (“erlotinib”) given 4-6 h before radiation. Experiments were repeated three times and average of results are shown. Survival fractions are plotted on a log-scale. Erlotinib treatment resulted in statistically significant reduction in cell survival of Dharma cells for radiation doses of 0, 2, 4 and 6 Gy, and statistically significant reduction in cell survival for D17 cells at 0 Gy, but did not promote enhancement of radiation effects for D17 or Abrams cell lines. *p < 0.05 indicates statistical significant reduction in clonogenic survival compared to control at the corresponding radiation dose

Erlotinib showed single agent activity through reduction in clonogenic survival in 2 out of 3 cell lines: *p* < 0.0001 for Dharma and *p* = 0.0003 for D17 (Fig. 1). No effect was seen in Abrams cells. Radiation administered at doses ranging from 2 to 10 Gy demonstrated a dose dependent reduction in clonogenic survival, as expected, in all 3 OSA cell lines examined (Fig. 2). Treatment with erlotinib four to six hours prior to radiation therapy resulted in a significant reduction in clonogenic survival of Dharma OSA cells for the lower radiation doses of 2 Gy (*p* < 0.0001), 4 Gy (*p* < 0.0001) and 6 Gy (*p* = 0.0127). This effect was lost at the higher radiation doses that resulted in a lower survival fraction from radiation treatment alone. In this cell line the shape of the survival curve for the “erlotinib” group had a more narrow shoulder compared to the “control” curve (Fig. 2), indicating the potential for reduced sublethal damage repair in these erlotinib treated cells. Enhancement of radiation effects was not observed in D17 (*p* = 0.39) and Abrams (*p* = 0.71) OSA cells.

### Effects of erlotinib and radiation on cell viability

**Fig. 3:**
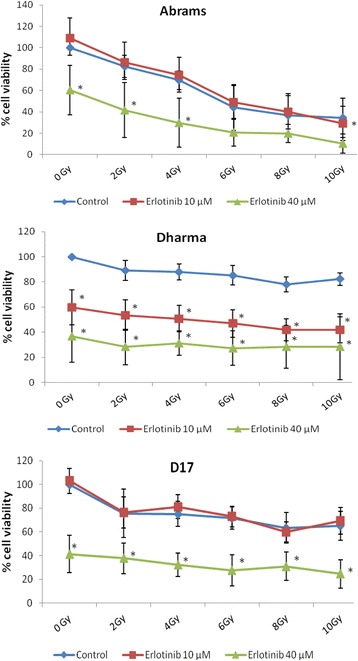
Cell viability assays 72 h post-radiation. Cells were treated with either radiation only (“control”), or radiation plus erlotinib at 10 μM or 40 μM. Experiments were repeated three times and average of results are shown. Addition of erlotinib resulted in statistically significant decreases in cell viability for Dharma cells at 10 μM, and for all cell lines at 40 μM dose. Enhancement of radiation effects were less pronounced at 10 μM, as seen in Dharma cells. *p <0.05 indicates statistically significant reduction in percentage of viable cells compared to control group at the corresponding radiation dose

Cell viability assays were assessed 72 h post-radiation (Fig. 3). Radiation administered at doses ranging from 2 to 10 Gy demonstrated dose dependent reductions in cell viability for 2 out of 3 OSA cell lines. The viability of Dharma cells was less impacted by radiation but interestingly, these cells were more sensitive to single agent erlotinib on cell viability assays, with statistically significant reductions in cell viability for all erlotinib treated groups (*p* < 0.0001), as shown in Fig. 3. Given the lack of response seen in 2 cell lines with erlotinib at 10 μM, a higher dose of 40 μM was tested. The viability of all 3 cell lines was reduced by this higher, but clinically/pharmacologically less relevant concentration of erlotinib. Addition of erlotinib at 40 μM resulted in decreased cell viability compared to radiation alone for all cell lines, however these effects were not statistically significant for Abrams cells at radiation doses above 4 Gy (*p* = 0.14). Treatment with erlotinib at the lower 10 μM dose further decreased viability in radiated Dharma cells (*p* ≤ 0.0002), but failed to provide enhancement of radiation effects for Abrams (*p* = 0.25) and D17 cells (*p* = 0.38).

### Expression of target proteins

**Fig. 4:**
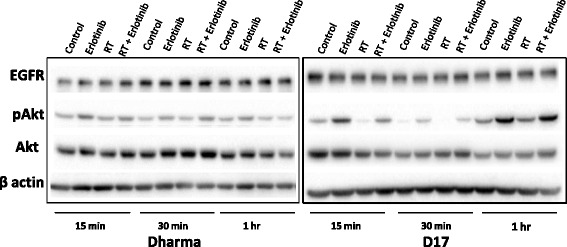
Western blot analysis of EGFR and downstream proteins. EGFR, total Akt and p-Akt were detected in all OSA cell lines investigated. Higher levels of p-Akt were seen after treatment with erlotinib, with or without radiation, in Dharma and D17 cells at 0.25, 0.5, 1, 2 and 24 hours

Western blot analyses detected endogenous expression of EGFR, total Akt and p-Akt in all three OSA cell lines investigated. Treatment with erlotinib, with or without radiation, increased levels of p-Akt in Dharma and D17 cells at 0.25, 0.5, 1, 2 and 24 h after radiation treatment (Fig. 4). Levels of p-Akt showed minimal variation among treatment groups in Abrams cells. Total Akt and EGFR were detected in all cell lines at all time points and treatment combinations, with no consistent variations seen among treatment groups.

### Effects of erlotinib and radiation on VEGF levels

**Fig. 5:**
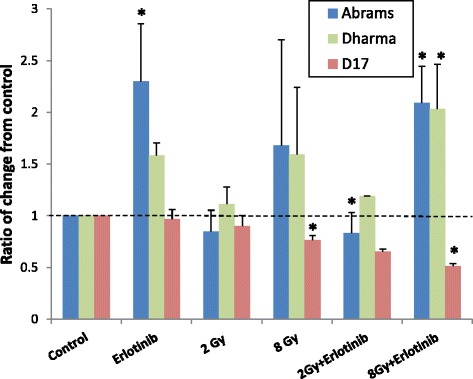
Concentration of VEGF in conditioned media 72 h post-radiation. VEGF levels are expressed as a ratio of change from control. *p < 0.05 indicates statistical significant change. Changes in VEGF levels were variable among cell lines, but significant changes occurred most consistently with combination RT plus erlotinib treatment

Secreted VEGF was detected in the conditioned media from all three canine OSA cell lines investigated (Table 1). Changes in VEGF levels compared to control occurred more consistently after combination treatment with radiation doses of 2 and 8 Gy (Fig. 5, Table 2). Interestingly, conditioned media from Dharma and Abrams cells showed increases in VEGF levels, whereas D17 cells showed decreases. Exposure to radiation at 8 Gy provided a significant reduction in VEGF levels for D17 cells (*p* < 0.09), but no other statistically significant changes were observed.

**Table 1:** Median VEGF concentration in conditioned media 72 h post-radiation (pg/mL)

	Abrams	Dharma	D17
Control	57.8 ± 36.4	476.7 ± 177.2	143.7 ± 60.1
Erlotinib	144.1 ± 63.4	413.9 ± 204.6	157.6 ± 91.4
2Gy	34.8 ± 20.4	465.8 ± 181.1	139.2 ± 57.1
8Gy	21.1 ± 7.7	447.3 ± 162.9	135.5 ± 37.8
2Gy + Erlotinib	130.4 ± 55.6	490.9 ± 225.3	148.9 ± 73.3
8Gy + Erlotinib	52.8 ± 15.9	398.8 ± 92	163.4 ± 54.9

**Table 2:** Median VEGF concentration 72 h post-radiation normalized to cell viability (pg/mL) * indicates significant change from control (*p* < 0.05)

	Abrams	Dharma	D17
Control	0.57	4.76	0.76
Erlotinib	1.22*	7.66	0.75
2Gy	0.37	5.22	0.61
8Gy	0.44	5.67	0.49*
2Gy + Erlotinib	1.32*	9.96	0.56
8Gy + Erlotinib	1.14*	9.32*	0.38*

## Discussion

The interaction of ionizing radiation with cells promotes both direct and indirect effects. Energy absorption can induce direct damage of molecules, however most of the energy deposited within cells is absorbed by water, generating free radicals. These are highly reactive molecules that can cause breakage of deoxyribonucleic acid (DNA) strands. If damaged DNA is not successfully repaired, either cell death or chromosomal aberrations may occur upon cell division [34]. With the exception of a few cell types, such as lymphocytes, that undergo apoptosis shortly after radiation exposure, most cell death secondary to irradiation takes place by mitotic catastrophe [34]. Rapidly proliferating cells have a high rate of cell division, and will therefore be more sensitive to radiation effects, or at least manifest the consequences of radiation damage sooner than slower dividing cell populations. However, cells that are proficient in DNA repair will be more resistant to radiation cytotoxicity.

After irradiation, cells may continue to be metabolically active (which is detectable in viability assays), but they may lose the capacity to undergo normal cell division and maintain continued reproductive ability [34]. Clonogenic survival assays after RT assess a cell’s ability to survive treatment, preserve cell division and repopulate the tumor, and therefore these assays provide an important in vitro assessment of potential therapeutic success. Radiation dose-response cell survival curves based on colony formation assays represent the total cumulative clonogenic outgrowth. The shoulders of these curves illustrate the capability of cells to repair sublethal DNA damage, and a wider shoulder indicates more efficient repair and subsequent repopulation, keeping clonogenic survival high. Cell survival curves in this current study were in agreement with previously reported RT dose-response curves for canine OSA cells, displaying a wide shoulder and moderate radioresistance [3]. Treatment with erlotinib provided statistically significant reductions in cell survival of Dharma cells at doses of 2, 4 and 6 Gy compared to control groups (Fig. 2). The radiosensitization effects of erlotinib are proposed to be, at least in part, secondary to cell cycle arrest in the G1 phase [4, 17, 18]. Cells in G1 phase are less radiosensitive than cells in G2 or M phases of the cell cycle, which makes radiosensitization by EGFR inhibitors appear counterintuitive. However, arrest in G1 also provides a cytostatic effect that prevents tumor cell repopulation between fractions of RT, still potentially enhancing the efficacy of a radiation protocol [4, 17, 18, 34]. It is possible that the sequence of treatment with a targeted EGFR inhibitor may play a role in radiosensitization. In this study cells were pre-treated with erlotinib 4 h prior to radiation, and drug treatment only after radiation was not investigated. Figure 2 also illustrates a narrowing of the shoulder in the cell survival curve for Dharma cells, indicating that decreased repopulation may have contributed to enhancement of radiation effects seen in this cell line. Colony formation assays in the present study showed no radiosensitizing effects of erlotinib on Abrams or D17 cells. Finally, it is also unknown whether Abrams cells, which appear to be more inherently resistant to erlotinib based on the results of Fig. 1, fail to undergo any G1 arrest after treatment with this drug.

Cell viability assays, such as the Resazurin assay, rely on bioreduction of the reagent dye by metabolically active cells, providing an indirect determination of cell viability. Such assays may not reflect the later death following cell divisions that is reflected in clonogenic survival assay results. Nevertheless, cell viability was assessed in the present study to investigate possible radiation enhancing effects of erlotinib by multiple mechanisms (Fig. 3). Abrams cells showed marked radiation dose-dependent reduction of cell viability, but moderate radioresistance on clonogenic survival assays. This discrepancy could potentially be explained by efficient repopulation. Abrams cells are very fast growing with a doubling time of approximately 17 h. Therefore at 72 h post-radiation, multiple cell divisions would likely have occurred, with consequent mitotic deaths, and corresponding low cell viability on Resazurin assays. Any surviving clones would have then undergone repopulation, resulting in the cell survival curves shown in Fig. 2. It could be concluded that erlotinib failed to prevent repopulation of Abrams cells, at least at the 10 μM dose. These findings were in contrast to Dharma cells, which showed less pronounced dose-dependent effects of radiation on cell viability assays, yet radiation demonstrated consistent suppression of clonogenic survival. Dharma cells have a doubling time of 34 h. Thus, after only 72 h the cytotoxic effects of RT may have been undetectable, as a significant proportion of cells have not yet undergone mitosis. D17 cells showed moderate radiation dose-response sensitivity on cell viability assays, which was more pronounced on clonogenic survival assays. Interestingly, the doubling time of D17 cells is 23 h, which is longer than Abrams cells but shorter than Dharma cells.

Erlotinib treatment promoted cytotoxic effects as a single agent at 10 μM for Dharma and D17 cells, and at 40 μM for all three cell lines investigated. Additionally, enhancement of radiation effects was seen at the 40 μM dose for all cell lines on cell viability assays. In addition to dosing, the order of treatment and period of exposure can influence the effects of combination therapy. In this study erlotinib was administered to cells 4 to 6 h prior to RT, and remained in the media until the end of experiments, in an effort to mimic how RTK inhibitors are used clinically. It is possible that a more prolonged period of erlotinib exposure prior to RT would have promoted enhancement of RT effects on D17 and Abrams cells. Nevertheless, the cytotoxic and radiation enhancing effects of erlotinib demonstrated in this current study support in vivo evaluation of EGFR inhibition as a possible treatment strategy for a subset of canine OSA cases. As erlotinib-induced enhancement of RT effects on cell viability were more pronounced at RT doses of 2 and 4 Gy, it can be expected that EGFR inhibition might be more effective in potentiating the effects of hyperfractionated curative protocols as opposed to the currently used palliative RT protocols. Further investigations of the ideal dosing, timing of drug exposure and RT protocol, utilizing additional OSA cells, xenograft models and other EGFR inhibitors is recommended to improve our understanding of potential radiosensitization effects of EGFR targeting in canine OSA. Given that not all patients are likely to benefit from this therapy, evaluation of EGFR expression and pathway activation for individual tumors could be investigated further as potential biomarkers of treatment response.

Protein analysis by Western blot confirmed EGFR expression in all three cell lines, with no variation in levels among treatment groups consistently throughout the time points examined. In contrast to antibody therapeutics such as cetuximab that can impact receptor trafficking, the small molecule kinase inhibitor erlotinib may not be expected to cause decreased total EGFR with signaling inhibition. The protein Akt was evaluated as a potential downstream indicator of EGFR kinase signaling inhibition. Activation of Akt post-radiation has been documented in human carcinoma and glioblastoma cells in vitro [28–30]. In the current study, increased levels of p-Akt post RT were not observed. It is possible that RT treatment does not activate the EGFR pathway in canine OSA cells as it occurs with human carcinoma and glioblastoma cells [28–30]. Interestingly however, increased levels of p-Akt were observed after erlotinib was used as a single agent or in combination with RT in D17 and Dharma cells. Increased levels of p-AKT may contribute to cell survival, and this was an unexpected finding with erlotinib treatment. These findings in the context of EGFR inhibitor use suggest that the EGFR pathway may not be exclusively responsible for the radioresistance of canine OSA, and illustrate that signaling responses after molecular targeting agents may be multifaceted. Other signaling cascades downstream of EGFR not investigated herein, such as the mitogen-activated protein kinases (MAPK/erk) pathway, could also be involved in the cytotoxic effects of erlotinib. Further evaluation of signaling events post RT and EGFR inhibition for canine OSA cells are warranted, as such studies could shed more light on the potential mechanisms involved in this treatment and improve targeted therapeutic strategies for this cancer.

The amount of VEGF secreted by OSA cells constitutively and after treatment with RT, erlotinib and combinations was quantified in this study. Increased serum VEGF levels in dogs with OSA has been correlated with decreased disease free intervals [35], and constitutive VEGF levels have previously been observed in canine OSA cells [33]. Dose-dependent increases in VEGF levels after RT have been documented in human glioblastoma cells and in lung cancer mouse xenografts [36, 37], and proposed to be associated with radioresistance. In the veterinary literature, RT up-regulated VEGF production in a melanoma cell line in a dose-dependent manner [38], but no changes in VEGF levels post- radiation were seen in a mast cell tumor cell line [39]. There are also correlations between the EGFR and VEGF pathways, as these share parallel and reciprocal downstream signaling mechanisms, and exert direct and indirect effects on tumor cells that contribute to cancer progression [40]. Additionally, epidermal growth factor, an important ligand for EGFR, also drives VEGF expression, and an overactive VEGF pathway plays a role in tumor resistance to treatment with EGFR inhibitors [40, 41]. Treatment with gefitinib, a selective EGFR RTK, resulted in decreased cell proliferation and decreased microvascular density and VEGF levels in murine renal cell carcinoma [42].

In the present study, VEGF production was not up-regulated after RT, and statistically significant decreased levels were seen by D17 cells after 8 Gy. Treatment with RTK inhibitors can modulate VEGF levels in an off-target manner. Increased VEGF levels have been found in vitro after canine OSA cells were treated with masitinib, a RTK inhibitor targeting c-Kit and platelet-derived growth factor receptor [33]. In our study, statistically significant changes in VEGF levels occurred more consistently after combination therapy. Additionally, D17 cells showed decreases whereas Dharma and Abrams cells had increases in VEGF production. This variability in VEGF levels post tyrosine kinase inhibitor treatment and RT illustrates the complexity of responses of individual cancers to cytotoxic stimuli, and the need for further investigation of angiogenic responses to anti-cancer therapeutics.

## Conclusions

Erlotinib treatment promoted modest enhancement of radiation effects in canine OSA cells, and showed activity as a single agent, indicating a possible role of EGFR inhibition in the treatment of a subset of OSA patients. Radioresistance of OSA cells does not appear to depend exclusively on EGFR signaling. Expanding research into signaling cascade alterations and angiogenic responses to combinations of RT with RTK inhibitors are worthy of further investigation.

## Abbreviations

Akt, Serine/threonine kinase, also known as protein kinase B; DNA, Deoxyribonucleic acid; EGFR, Epidermal growth factor receptor; MAPK, mitogen-activated protein kinase, also known as erk; NSCLC, Non-small-cell lung cancer; OSA, Osteosarcoma. p-Akt, Phosphorylated Akt; PI3K, Phosphatidylinositol-3-kinase; PIP3, Phosphatidylinositol-3,4,5-trisphosphate; RT, Radiation therapy; RTK, Receptor tyrosine kinase; SCC, Squamous cell carcinoma; VEGF, Vascular endothelial growth factor
